# T219. THE ROLE OF MELATONIN AND MELATONIN AGONISTS IN COUNTERACTING ANTIPSYCHOTIC-INDUCED METABOLIC SIDE EFFECTS: A SYSTEMATIC REVIEW

**DOI:** 10.1093/schbul/sby016.495

**Published:** 2018-04-01

**Authors:** Won-Myong Bahk, Young Joon Kwon, Bo-Hyun Yoon, Sang-Yeol Lee, Kwanghun Lee, Duk-In Jon, Moon Doo Kim, Eunsung Lim

**Affiliations:** 1 Catholic University of Korea; 2 Soonchunhyang University; 3 Naju National Hospital; 4 Wonkwang University, School of Medicine; 5 College of Medicine, Dongguk University; 6 College of Medicine, Hallym University; 7 School of Medicine, Jeju National University; 8 Shinsegae Hospital

## Abstract

**Background:**

Melatonin administration to high cholesterol-treated and high fat-treated rats has been shown to suppress body weight and visceral adiposity. In addition, in various animal models related to obesity, metabolic syndrome, and diabetes, melatonin has beneficial efficacy in ameliorating various metabolic symptoms, including attenuating weight gain, lowering blood pressure (BP), and improving insulin resistance. This systematic review aims to investigate whether melatonin or melatonin agonists significantly attenuate metabolic side effects among psychiatric populations treated with atypical antipsychotics.

**Methods:**

Four randomized controlled trials were identified through a comprehensive literature search using MEDLINE, EMBASE, and the Cochrane Library on 22 October 2015. These four trials (including three melatonin studies and one ramelteon study) included 138 patients, of whom 71 were treated with melatonin or ramelteon and 67 were treated with a placebo. Because of high heterogeneity, we did not carry out a meta analysis.

**Results:**

Melatonin was beneficial in lowering blood pressure among bipolar disorder patients; this blood pressure-lowering effect was not prominent among schizophrenic patients. Melatonin appeared to improve lipid profiles and body composition and attenuated weight gain among both schizophrenic and bipolar disorder patients. Ramelteon showed a significant efficacy in lowering total cholesterol level.

**Discussion:**

Despite the few studies included, this systematic review provided promising evidence of the potential benefits of melatonin and its agonists in attenuating one or more components of metabolic syndrome among psychiatric patients using atypical antipsychotics.

